# Encephalitis associated with human herpesvirus-7 infection in an immunocompetent adult

**DOI:** 10.1186/s12985-017-0764-y

**Published:** 2017-05-25

**Authors:** Mónica Parra, Adoración Alcala, Cristina Amoros, Anna Baeza, Antonio Galiana, David Tarragó, Miguel Ángel García-Quesada, Victoria Sánchez-Hellín

**Affiliations:** 1 Department of Microbiology, Hospital General Universitario de Elche, Camino Almazara 11, 03203 Elche, Spain; 2 Department of Intensive Medicine, Hospital General Universitario de Elche, Elche, Spain; 3 Institute of Health Carlos III, National Centre for Microbiology, Madrid, Spain; 4 Department of Neurology, Hospital General Universitario de Elche, Elche, Spain

**Keywords:** *Human herpesvirus-7* encephalitis, Immunocompetent adults, Clinical significance HHV-7 CNS infection

## Abstract

**Background:**

Primary Human herpesvirus-7 (HHV-7) infection usually occurs during childhood and causes several clinical manifestations: mainly exanthem subitum (*roseola infantum*), followed by a lifelong latent state with possible reactivation in case of immunodeficiency. Nevertheless, some considerably different approaches exist regarding the natural history of HHV-7 and the possible consequences of HHV-7 infection in immunocompetent adults. In particular, little is known about its pathogenic role in central nervous system (CNS) disease in nonimmunosuppressed adults. Specifically, in case of encephalitis, it is important to distinguish between infectious encephalitis and postinfectious encephalomyelitis for the management of patients

**Case presentation:**

We describe here a case of encephalitis associated to human herpesvirus-7 with associated polymyeloradiculopathy in an immunocompetent patient which may contribute to the delineation of the approach to a patient profile with a similar clinical presentation and evolution to those presented in the literature.

**Conclusions:**

This case may alert clinicians to consider this specific etiology in the differential diagnosis of encephalopathy in patients with suspected infectious encephalitis who do not respond to acyclovir or in patients who develop acute polymyeloradiculopathy, considering that HHV-7 may be a pathological factor and that a timely diagnosis is crucial for the early administration of specific treatment.

**Electronic supplementary material:**

The online version of this article (doi:10.1186/s12985-017-0764-y) contains supplementary material, which is available to authorized users.

## Background

Human herpesvirus-7 (HHV-7) is a ubiquitous virus that belongs to the subfamily of β-herpesviruses (together with cytomegalovirus and human herpesvirus-6). Primary HHV-7 infection usually occurs during childhood and may cause several clinical manifestations: mainly exanthem subitum (*roseola infantum*), followed by a lifelong latent state with possible reactivation in case of immunodeficiency [1]. Nevertheless, some considerably different approaches exist regarding the natural history of this infectious agent and the possible consequences of HHV-7 infection in immunocompetent adults, pertaining both to late primary HHV-7 infection or reactivation of the virus from macrophages and/or CD4+ T cells. In particular, little is known about its pathogenic role in central nervous system (CNS) disease in nonimmunosuppressed adults. Specifically, in case of encephalitis, it is important to distinguish between infectious encephalitis and postinfectious encephalomyelitis for the management of patients. We describe here a case of HHV-7 encephalitis with associated polymyeloradiculopathy in an immunocompetent patient.

## Case presentation

A 26-year-old immunocompetent male presented with dizziness, blurred vision, a 3-day history of fever, frontal headache, cervical and lumbar pain, and vomiting during the last 24 h. There was no significant personal history apart from animal exposure: contact with dogs and cats several days before the onset of symptoms and the presence of a tick on his back (information provided by his relatives). Physical examination revealed the presence of somnolence. On admission, the patient presented cranial nerve affectation (ophthalmoplegia), vertical nystagmus in attempted supra-version, mitigated osteotendinous reflexes, indifferent bilateral superficial cutaneous reflexes, and dysmetria, which was observed on the finger-to-nose test. A cerebrospinal fluid (CFS) sample contained 110 leucocytes/μl (lymphocytes + monocytes) and 100 red blood cells/μl, 82 mg/dl of protein, and 60 mg/dl of glucose (blood glucose, 108 mg/dl). A second CFS sample obtained 3 days after the collection of the first one contained 200 leucocytes/μl (95% monocytes), 61 mg/dl of protein, and 54 mg/dl of glucose (blood glucose, 88 mg/dl). Progressive deterioration of the level of consciousness with a Glasgow Coma Score (GCS) of 8 (V1, O1, M6) led to a requirement for mechanical ventilation.

The patient was empirically treated with acyclovir, ceftriaxone, ampicillin, doxycycline, and corticoids. His level of consciousness improved (GCS 11 [O4, V1, M6]), although the ophthalmoplegia persisted. Electroencephalography (EEG) showed irregular slow activity with slow bilateral waves in the temporal and frontal areas, which was consistent with encephalitis. Magnetic resonance imaging (MRI) of the brain was normal. Normal findings were obtained for a wide spectrum of antibodies (anti-Jo-1, anti-Sm, anti-SS-A, anti-SS-B, anti-nRNP, anti-Scl-70, anti-c-ANCA, anti-p-ANCA, anti-dsDNA, anti-amphiphysin, anti-CV2, anti-PNMA2, anti-Yo (PCCA-1), anti-Hu (ANNA-1), anti-RI (ANNA-2), and anti-ganglioside). On the 5th evolution day, he developed flaccid paralysis of his right arm and lower limbs with abolishment of osteotendinous reflexes. Electromyography (EMG) performed on the 8th evolution day revealed an F-wave alteration in the upper and lower limbs that was indicative of the initial stage of polyradiculoneuritis. Cerebrospinal fluid (CFS), serum, and whole-blood samples were screened for the identification of possible etiologies. Follow-up MRI investigations performed in our patient revealed the presence of cervical and dorsolumbar myelitis. Intravenous immunoglobulins were administered to the patient, who was considered as having acute polymyeloradiculopathy. After treatment, the ophthalmoplegia disappeared, the strength of the upper-right limb was recovered, and the strength of the lower limbs was improved. The patient was subsequently discharged; however, urinary incontinence persisted. Four months later, the patient had recovered completely.

The results of the microbiological assessment, according to clinical and risk factors were as follows. First-line investigation: on day 1; gram stain and bacterial culture, alpha-herpesvirus (*herpes simplex virus* 1, *herpes simplex virus* 2, and *varicella-zoster virus*), and enterovirus were negative. On day 3; *Treponema pallidum* and *Cryptococcal neoformans* (serum and CFS), *Mycoplasma pneumoniae*, *Epstein–Barr virus*, and *Cytomegalovirus* were negative. Because of animal exposure and insect contact, *Borrelia burgdorferi*, *Borrelia* spp., *Rickettsia* spp., *Bartonella*, *Coxiella burnetii*, *Anaplasma/Ehrlichia*, *Brucella*, and *Toxoplasma* (serum and CFS) tests were performed and were negative. Screening for HIV (serology and RNA), hepatitis A, B, and C, and *Mycobacterium tuberculosis* (CSF) was also negative. Second-line investigation: on day 4; *West Nile*, *Toscana*, *Choriomeningitis*, *Yellow fever*, *Dengue*, and *Chikungunya* arbovirus were negative. Third-line investigation: on day 10, because of the deterioration of the patient and the lack of an etiologic cause for the symptoms, alpha-herpesvirus and enterovirus were screened again, with negative results. In addition, as vector-borne disease was the most likely explanation for the patient’s disease, on day 14 (early convalescent phase) he was tested for *Borrelia burgdorferi*, *Rickettsia conorii*, and *Coxiella burnetii* (serum and whole blood), as well as *Rickettsia* spp., *Bartonella*, *Coxiella burnetii*, and *Anaplasma/Ehrlichia* (whole blood; frozen on day 7 [acute phase]) in parallel, with negative results. Finally, *human herpesvirus*-6, HHV-7, and *human herpesvirus*-8 were suspected in the absence of positive outcomes and presence of deterioration despite treatment with acyclovir. HHV-7 DNA was detected in the first CSF sample by Multiplex Nested PCR, which detects DNA from HHV-6, HHV-7, and HHV-8 on day 18 [2]. Fluorescent anti-HHV-7 IgG testing in serum samples on day 3 and days 14 and 21 revealed an increase in anti-HHV-7 titers from 1:32 to 1:64, in a retrospective analysis. However, HHV-7 antibody avidity testing, to determine primary infection vs reactivation of HHV-7 in the nervous system, could not be performed. Details of the microbiological assessment performed on the patient’s samples are shown in the Additional file 1: Table S1. Acyclovir was discontinued after microbiological diagnosis and the patient was treated with ganciclovir over 3 weeks.

## Discussion

Our approach to this patient included a differential diagnosis that excluded noninfectious CNS diseases such as structural lesions, brain abscesses, parameningeal infections, and metabolic and toxic encephalopathies; however, a definitive diagnosis could not be established that discriminated between HHV-7 encephalitis or postinfectious encephalomyelitis. The detection of HHV-7 DNA in the patient’s CSF concomitant with neurological disease in association with the exclusion of all alternative etiological causes (according to the clinical practice guidelines of the Infectious Diseases Society of America [3]) supported HHV-7 as the possible cause of the encephalitis, based on the criteria that were established by Schwartz et al. [4]. As a consequence of antibody avidity could not be determined in our laboratory to differentiate between primary and past infection, we can not certainly establish whether encephalitis associated to HHV-7 infection in our patient was secondary to late primary infection or reactivation. Conversely, HHV-7 is a virus that is highly prevalent and has been detected in normal brain tissue [5]. Therefore, CSF PCR has a low positive predictive value. In fact, the patient had already improved before ganciclovir therapy was administered. Thus, a combination of HHV-7 primoinfection or reactivation and immune response is believed to have contributed to the development of encephalomyelitis. It is likely that HHV-7 was the infecting microorganism that was responsible for the immunological response, taking into account our patient’s clinical improvement after treatment with intravenous immunoglobulin therapy.

The case presented here contributes to the delineation of the approach to a patient profile with a similar clinical presentation and evolution to those presented in the literature [6–11]. All relevant information about these cases is presented in Table 1. In our case, despite the administration of empirical antiviral therapy with acyclovir as the first line for viral encephalitis, the patient developed polymyeloradiculopathy consisting of urinary retention, respiratory failure, and flaccid paralysis of the limbs, progressing to quadriplegia. Notably, these findings are similar to those of the HHV-7 encephalitis cases published previously, which occurred in both immunocompetent and nonimmunocompetent adults; this may alert clinicians to consider this specific etiology.

**Table 1:** Clinical features, diagnosis, and treatment of similar cases reported in the literature

Clinical diagnosis/Immune status	Age	Gender	Presentation	Evolution before treatment	Microbiological HHV-7 diagnosis	CSF	Primoinfection vs reactivation	Specific Treatment	Reference
Encephalitis and flaccid paralysis/Immunocompetent	19	Male	Severe headache, vomiting, dizziness, and urinary retention	Ataxia, impaired pupillary reflexes, reduced level of consciousness, and flaccid paralysis of the lower limbs progressing to quadriparesis	HHV-7 DNA in CSF (PCR [12]) and low avidity HHV-7 IgG	65 leucocytes/μl (63 lymphocytes + 2 neutrophils) and 2 red blood cells/μl, elevated protein of 80 mg/dl, and 48,6 mg/dl of glucose (blood glucose 90 mg/dl)	P	-	Ward KN 2002 [6]
Acute myeloradiculopathy/Immunocompetent	26	Male	Progressive motor weakness, tingling in the extremities, and dysphasia	Cranial nerve involvement and autonomic dysfunctions	HHV-7 DNA in CSF by real time-PCR and increase in anti-HHV-7 titers from 1:16 to 1:64 (IFA)	Normal at admission with an increase in protein of 89 mg/dl and a modest pleocytosis (8 cells/μl) by day 20.	R	Intravenous immunoglobulin (400 mg/kg/day) for 5 days	Mihara T 2005 [8]
Encephaloradiculomyelitis associated to HHV-7and CMV co-infection/Immunocompetent	51	Male	Fever and gradual loss of strength in lower limbs	Disorientation and confusion, flaccid paraplegia, urinary retention, and constipation	HHV-7 DNA in CSF (nested PCR)^a^ and anti-HHV-7 IgG but not IgM in serum (IFA)	Pleocytosis (250 cells/μl) with 93% lymphocytes, 10 red blood cells/μl, high protein levels (950 mg/dl) and normal glucose.	R	Ganciclovir and dexamethasone	Ginanneschi F 2007 [9]
Acute myelitis/Immunocompetent	34	Male	Fever, urinary retention, and weakness in the lower limbs	-	HHV-7 RNA in CSF (PCR-Microarray, Entherpex®, Genomica)	35 leucocytes/μl (predominantly monocytes) elevated protein of 75 mg/dl.	-	Methylprednisolone (1 g/day) and ganciclovir	Miranda M 2011 [10]
Neurological disorder/Immunocompetent	27	Male	Persistent headache and fever	-	HHV-7 RNA in CSF (PCR-Microarray, Entherpex®, Genomica)	160 leucocytes/μl (predominantly monocytes) elevated protein of 75 mg/dl	-	-	Miranda M 2011 [10]
Encephalitis with polymyeloradiculopathy/Immunocompetent	26	Male	Headache and fever, dizziness, blurred vision, cervical and lumbar pain, and somnolence	Flaccid paralysis of right arm and lower limbs appeared on 5th evolution day	HHV-7 DNA in CSF (Multiplex Nested PCR)	110 leucocytes/μl (lymphocytes + monocytes) and 100 red blood cells/μl, elevated protein of 82 mg/dl, and 60 mg/dl of glucose (blood glucose, 108 mg/dl)	P or R or PE	Intravenous immunoglobulin Ganciclovir	Current case
Acute myelitis/Bone marrow recipient	47	Male	Pyrexia and urinary retention	Spastic paraparesis	HHV-7 DNA in CSF (PCR [12]) and high avidity HHV-7 IgG before transplant	48 leucocytes/μl (predominantly lymphocytes), 235 red blood cells/μl, elevated protein of 1.22 g/l, and 52.2 mg/dl of glucose (blood glucose mg/dl 99)	R	Methylprednisolone (1 g/day)	Ward KN 2002 [7]
Acute myelitis/HIV-infected	40	Male	Asymmetric paresthesia and hypoesthesia in lower limbs	Flaccid paraparesis and severe lower-limb hypoesthesia	HHV-7 DNA in CSF (PCR-Microarray, Clart® Entherpex, Genomica)	1 lymphocyte, 0 erythrocytes, 25,88 mg/dl proteins and 59 mg/dl glucose	P or R	Foscarnet	Escobar-Villalba A 2016 [11]

^a^Nested PCR performed on CSF sample was also positive for cytomegalovirus (CMV); IFA, indirect immunofluorescence assay; P: Primoinfection; R: Reactivation; PE: Postinfectious encephalomyelitis

## Conclusions

It may be concluded, regarding the importance of the confirmed laboratory diagnosis, that HHV-7 should be considered in the differential diagnosis of encephalopathy in patients with suspected infectious encephalitis who do not respond to acyclovir or in patients who develop acute polymyeloradiculopathy, considering that HHV-7 may be a pathological factor and that a timely diagnosis is crucial for the early administration of specific treatment.

## Additional file

Additional file 1: Table S1. Microbiological assessment performed on the patient’s samples. (ODT 19 kb)
