# Supplementary material for: Encephalitis associated with human herpesvirus-7 infection in an immunocompetent adult
Source: Virol J. 2017 May 25;14:97. doi: 10.1186/s12985-017-0764-y (PMC5445330; doi:10.1186/s12985-017-0764-y)
Supplement: Supplementary file 1 [file 12985_2017_764_MOESM1_ESM.odt]

Table S1. Microbiological assessment performed on the patient’s samples 

| Specimen type, etiologic agent | Test type | Criteria for testing | Time | Result |
| --- | --- | --- | --- | --- |
| CFS |  |  |  |  |
| Gram stain and bacterial isolation | Culture | First-line investigation | Day 1 | - |
| α-herpesvirusa | Real-time PCR | First-line investigation | Day 1 | - |
| Enterovirus | Real-time PCR | First-line investigation | Day 1 | - |
| Cryptococcus neoformans | Antigen | First-line investigation | Day 3 | - |
| Treponema pallidum | VDRL | First-line investigation | Day 3 | - |
| Borrelia burgdorferi | IgG EIA | Animal exposureb | Day 3 | - |
| Toxoplasma gondii | IgG EIA | Animal exposureb | Day 3 | - |
| Brucella | Rose Bengal | Animal exposureb | Day 3 | - |
| West Nile virus* | Real-time PCR | Second-line investigation | Day 4 | - |
| Toscana virus* | Real-time PCR | Second-line investigation | Day 4 | - |
| Choriomeningitis virus* | Conventional PCR | Second-line investigation | Day 4 | - |
| HHV-6, HHV-7, HHV-8* | Multiplex NestedPCR | Third-line investigationc,d | Day 18 | - /+/- |
| Serum |  |  |  |  |
| Acute phase |  |  |  |  |
| HIV | EIA, PCR (HIV RNA) | First-line investigation | Day 3, 4 | -/- |
| Hepatitis B, C,  A | EIA | First-line investigation | Day 3 | - |
| Cryptococcus neoformans | Antigen | First-line investigation | Day 3 | - |
| Treponema pallidum | RPR | First-line investigation | Day 3 | - |
| Epstein-Barr virus | Paul bunnell, VCA IgM, VCA IgG | First-line investigation | Day 3 | -/-/+ |
| Cytomegalovirus | IgM, IgG EIA | First-line investigation | Day 3 | -/+ |
| Mycoplama pneumoniae | IgM EIA | First-line investigation | Day 3 | - |
| Borrelia burgdorferi | IgG, IgM EIA | Animal exposureb | Day 3 | - |
| Toxoplasma gondii | IgM, IgG EIA | Animal exposureb | Day 3 | -/- |
| Brucella | Rose Bengal | Animal exposureb | Day 3 | - |
| Coxiella burnetii | IgM, IgA, IgG IFA | Animal exposureb | Day 3 | -/-/- |
| Rickettsia conorii* | IgM, Total Antibodies IFA | Animal exposureb | Day 4 | -/+ [1/40] |
| West Nile virus* | IgM, IgG EIA | Second-line investigation | Day 4 | -/- |
| Toscana virus* | IgM, IgG EIA | Second-line investigation | Day 4 | -/- |
| Choriomeningitis virus* | IgM, IgG IFA | Second-line investigation | Day 4 | -/- |
| Chikungunya virus* | IgM, IgG IFA | Second-line investigation | Day 4 | -/- |
| Dengue virus* | IgM, IgG EIA | Second-line investigation | Day 4 | -/- |
| Coxiella burnetii | IgM, IgA, IgG IFA | Animal exposureb | Day 4 | -/-/- |
| Bartonella henselae* | IgM, IgG IFA | Animal exposureb | Day 4 | -/- |
| Bartonella quintana* | IgM, IgG IFA | Animal exposureb | Day 4 | -/- |
| α-herpesvirusa | Real-time PCR | Third-line investigationc | Day 10 | - |
| Enterovirus | Real-time PCR | Third-line investigationc | Day 10 | - |
| HHV-6, HHV-7, HHV-8* | Multiplex Nested PCR | Third-line investigationc,d | Day 21 | -/-/- |
| HHV-7 | IgG IFA | Confirmed HHV-7 disease | - | 1/320 |
| Early convalescent phase |  |  |  |  |
| Rickettsia conorii* | IgM, Total Antibodies IFA | Animal exposureb | Day 14 | -/- |
| Coxiella burnetii | IgM, IgA, IgG IFA | Animal exposureb | Day 14 | -/-/- |
| Borrelia burgdorferi | IgG, IgM EIA | Animal exposureb | Day 14 | -/- |
| Late convalescent phase |  |  |  |  |
| Borrelia burgdorferi | IgG, IgM EIA | Fourth-line investigatione,f | Day 28 | -/- |
| Leptospira* | IgM EIA | Fourth-line investigationg | Day 28 | - |
| HHV-7 | IgG IFA | Confirmed HHV-7 disease | - | 1/640 |
| Whole blood |  |  |  |  |
| Acute phase |  |  |  |  |
| Anaplasma / Ehrlichia* | Conventional PCR | Animal exposureb | Day 7 | - |
| Bartonella spp.* | Conventional PCR | Animal exposureb | Day 7 | - |
| Coxiella burnetii* | Real-time PCR | Animal exposureb | Day 7 | - |
| Rickettsia spp.* | Conventional PCR | Animal exposureb | Day 7 | - |
| HHV-6, HHV-7, HHV-8* | Multiplex Nested PCR | Third-line investigationc,d | Day 21 | -/-/- |
| Early convalescent phase |  |  |  |  |
| Rickettsia spp.* | Conventional PCR | Animal exposureb,f | Day 14 | - |
| Borrelia spp.* | Conventional PCR | Animal exposureb,f | Day 14 | - |
| HHV-6, HHV-7, HHV-8* | Multiplex Nested PCR | Third-line investigationc | Day 21 | -/-/- |

NOTE. CSF, cerebrospinal fluid; VDRL, venereal disease research laboratory; RPR, rapid plasma reagin; VCA, viral capsid antigen; PCR, polymerase chain reaction; IFA, indirect immunofluorescence assay; EIA, enzyme immunoassay; HIV, human immunodeficiency virus; HHV-6, human herpesvirus-6; HHV-7, human herpesvirus-7; HHV-8, human herpesvirus-8.

a HSV-1, herpes simplex virus type 1; HSV-2, herpes simplex virus type 2; and VZV, varicella-zoster virus.

b Contact with dogs and cats and presence of a tick on the back.

c Deterioration of the patient and no etiologic cause identified.

d Lack of response to acyclovir.

e Area of high prevalence.

f Late antibody response.

g Other investigations are negative.

*Processed at the Institute of Health Carlos III, National Centre for Microbiology, Spain.
